# Key Determinants of Health-Related Quality of Life Among Advanced Lung Cancer Patients: A Qualitative Study in Belgium and Italy

**DOI:** 10.3389/fphar.2021.710518

**Published:** 2021-09-23

**Authors:** Rosanne Janssens, Reinhard Arnou, Elise Schoefs, Serena Petrocchi, Clizia Cincidda, Giulia Ongaro, Serena Oliveri, Meredith Y. Smith, Evelyne Louis, Marie Vandevelde, Kristiaan Nackaerts, Gabriella Pravettoni, Isabelle Huys

**Affiliations:** ^1^ Department of Pharmaceutical and Pharmacological Sciences, KU Leuven, Leuven, Belgium; ^2^ Applied Research Division for Cognitive and Psychological Science, IEO, European Institute of Oncology IRCCS, Milan, Italy; ^3^ Department of Oncology and Hemato-Oncology, University of Milan, Milan, Italy; ^4^ Alexion Pharmaceuticals, Inc., Boston, MA, United States; ^5^ University of Southern California School of Pharmacy, Los Angeles, CA, United States; ^6^ Department of Pulmonology/Respiratory Oncology, University Hospital Leuven, Leuven, Belgium

**Keywords:** patient preferences, lung cancer, health-related quality of life, qualitative research, focus group discussions, patient-reported outcome (PRO), drug development, patient-relevant treatment outcomes

## Abstract

**Background:** The lung cancer (LC) treatment landscape has drastically expanded with the arrival of immunotherapy and targeted therapy. This new variety of treatment options, each with its own characteristics, raises uncertainty regarding the key aspects affecting patients’ health-related quality of life (HRQL). The present qualitative study aimed to investigate how LC patients perceive their HRQL and the factors that they consider to be most influential in determining their HRQL.

**Methods:** This qualitative research incorporates four focus group discussions, with six LC patients in each group. In total, 24 stage III and IV LC patients were included in the discussions, with Italian (*n* = 12) and Belgian (*n* = 12) patients, age range: 42–78, median age = 62 (IQR = 9.3 years), SD = 8.5; 62% men. Using thematic analysis, transcripts and notes from the FGDs were analyzed using NVivo software (edition 12).

**Results:** Three main themes capturing determinants of HRQL were identified. First, patients agreed on the importance of physical aspects (symptoms and side-effects) in determining their HRQL. In particular, skin conditions, nausea, fatigue, risk of infections, sensory abnormalities, pain, and changes in physical appearance were highlighted. Second, patients worried about psychological aspects, negatively impacting their wellbeing such as uncertainties regarding their future health state, and a lower degree of autonomy and independence. Third, patients underlined the importance of social aspects, such as communication with healthcare providers and social interaction with friends, family and peers.

**Conclusion:** This study demonstrates that physical, psychological, and social aspects are key factors driving LC patients’ HRQL. Gaining a better understanding of how LC patients perceive their HRQL and how it is affected by their illness and therapy will aid patient-centric decision-making across the drug life cycle, by providing stakeholders (drug developers, regulators, reimbursement bodies, and clinicians) insights about the treatment and disease aspects of importance to LC patients as well as the unmet needs LC patients may have regarding available treatment modalities. Finally, this study underscores a need for individual treatment decision-making that is considerate of uncertainties among LC patients about their future health state, and ways for improving communication between healthcare providers and patients to do so.

## Introduction

*Lung cancer* (LC) is the leading cause of cancer mortality due to its high incidence and low survival rate. With 2.09 million new cases and 1.76 million deaths in 2018 worldwide, LC is the deadliest cancer in men and second in women (Bray et al., 2018). *Non-small cell lung cancer* (NSCLC) is the most prevalent type of LC, accounting for 85–90% of all LC cases worldwide (Remon et al., 2020). With the emergence of innovative treatment modalities over the past decade, the LC treatment landscape has changed dramatically, with the range of options now extending beyond well-established therapies such as surgery, radiotherapy, and chemotherapy to include such new regimens such as targeted therapy, immunotherapy, and chemoimmunotherapy (Dong et al., 2019; Remon et al., 2020). LC treatments in development and use today differ in terms of benefits (e.g., in terms of progression-free survival, overall survival, response rate, and long-term benefits), side-effects (e.g., pain, nausea, vomiting, breathing problems, fatigue, physical changes such as weight changes, bleeding, hair loss, and uncertain long-term safety), psychological impact (e.g., emotional distress, affective disorders), route of administration, and treatment schedule (Dong et al., 2019; King-Kallimanis et al., 2019; Van Der Weijst et al., 2019; Remon et al., 2020).

While recent developments have resulted in a greater range of treatment options for NSCLC patients, the variety of LC treatment options and their associated characteristics also raises uncertainty regarding the key treatment and disease aspects affecting LC patients’ *health-related quality of life* (HRQL) (Blinman et al., 2010; Maric et al., 2010; Grassi et al., 2017). HRQL has been defined by the *US Food and Drug Administration* (FDA) as *“a multi-domain concept that represents the patient’s general perception of the effect of illness and treatment on physical, psychological, and social aspects of [his/her] life”* (US Food and Drug Administration, 2006). The variety of LC treatments, their characteristics, and the ascendency of patient-centered care require an informed decision-making by stakeholders involved in the medicinal product development, evaluation, and prescription that involves the elicitation and consideration of patient preferences (Marzorati and Pravettoni, 2017). As noted in prior research, patient preferences represent a crucial consideration for both clinical decision-making by healthcare providers, as well as decision-making by pharmaceutical companies, regulators, *Health Technology Assessment* (HTA) bodies, payers, and across the medicinal product life cycle (Petrocchi et al., 2021; The International Council for Harmonisation of Technical Requirements for Pharmaceuticals for Human Use (ICH), 2020; Janssens et al., 2019a; Janssens et al., 2019b; Soekhai et al., 2019; van Overbeeke et al., 2019a; van Overbeeke et al., 2019b; Whichello et al., 2019).

One way of determining what matters to patients is via performing a patient preference study. Patient preference studies use qualitative and/or quantitative methods to identify which treatment characteristics are important to patients, how important, and which tradeoffs patients are willing to make between various characteristics (The International Council for Harmonisation of Technical Requirements for Pharmaceuticals for Human Use (ICH), 2020; Patient Preference Information–Voluntary Submission, 2016; Patient-Focused Drug Development, 2018). In doing so, such studies illuminate key aspects affecting patients’ quality of life. Eliciting preferences from NSCLC patients is especially valuable in view of uncertainties regarding the impact of different (novel) LC treatments’ effects on patients’ lives, attitudes, and choices towards treatments. In addition, the FDA emphasizes that patient preference information is especially valuable in “preference sensitive situations”, i.e., situations where: 1) multiple treatment options exist and there is no option that is clearly superior for all patients, 2) the evidence supporting one option over others is considerably uncertain or variable, and 3) patients’ views about the most important benefits and acceptable risks of a technology vary considerably within a population and may differ from those of healthcare professionals (US Food and Drug Administration, 2016). Decision-making regarding the development, market approval, and reimbursement of new NSCLC treatments is therefore a preference sensitive situation, as such decision-making may depend on the preferences of patients for these diverse treatment characteristics (Blinman et al., 2010; Marzorati and Pravettoni, 2017; Petrocchi et al., 2021).

Previous empirical preference studies among LC patients were mostly quantitative in nature and have focused on chemotherapy (Hirose et al., 2005; Hirose et al., 2009; Blinman et al., 2010; Schmidt et al., 2016; Schmidt et al., 2017; Sugitani et al., 2020). This contrasts with the added value that qualitative methods provide; qualitative methods provide in-depth and meaningful information from patients, and hence, their use is recommended for understanding what matters most to patients, and why. Furthermore, qualitative methods with patients reduce the potential for misspecification of aspects most important for patients, for inclusion in drug development and evaluation, and thereby avoid overreliance on the views of experts and researchers (Coast et al., 2012). In doing so, using qualitative research for understanding what matters most to patients in relation to their HRQL, the data collected on patient perspectives and preferences is likely to be more comprehensive, meaningful, and a valid interpretation of the true patient perspective (47). Therefore, the present qualitative study aimed to investigate how LC patients perceive their HRQL and what LC patients consider to be most important in determining their HRQL, thereby expanding the body of evidence regarding LC patient preferences.

## Methods

### Study Context and Design

This study was conducted as part of the *Patient Preferences in Benefit-Risk Assessments during the Drug Life Cycle* (PREFER) project. The PREFER project’s objective is to develop evidence-based recommendations to inform stakeholders on how to conduct patient preference studies and how their results can be implemented in the drug decision-making process (PREFER, 2020). The present paper presents a secondary data analysis of *focus group discussions* (FGDs) with LC patients. A primary analysis of the discussions, describing overarching themes of treatment features of importance to LC patients has been published elsewhere (Petrocchi et al., 2021), as well as detailed information regarding the applied qualitative methodologies and limitations (Durosini et al., 2021). However, a specific and in-depth analysis of how LC patients perceive their HRQL, and what they consider to be the treatment- and disease-related factors influencing their HRQL was out of scope in the abovementioned papers. Therefore, the present paper provides a further analysis of cross-country HRQL related themes and detailed insights into the opinions from Italian and Belgian patients with stage III and IV NSCLC.

### Participant Recruitment

Participants were recruited in Italy and Belgium to allow for an understanding of which treatment aspects influencing HRQL were important for LC patients with differing patient characteristics, backgrounds, and who lived in countries with different kinds of healthcare systems (e.g., in terms of financing, service provision, and access to healthcare). Participants were purposely recruited between September 2019 and October 2019 by the treating oncologists at the Thoracic Oncology Division of the European Institute of Oncology in Milan (Italy) and the Respiratory Oncology Department of the University Hospitals in Leuven (Belgium). In Belgium, the *“Ethische Commissie Onderzoek UZ/KU Leuven”'* approved the study (reference S63007). In Italy, the *“Ethical Committee of the European Institute of Oncology IRCCS IEO”* approved the study (reference 1,027/19-IEO 1093).

Inclusion and exclusion criteria for the FGDs were defined and described in the protocol paper (Durosini et al., 2021). In particular, the following inclusion criteria were applied: 1) in treatment patients with a histological or cytological diagnosis of NSCLC stage III or IV as classified by the Union for International Cancer Control TNM Classification of Malignant Tumors (UICC TNM VIII Edition); 2) adult patients (≥18 years old). Stage III or IV patients were eligible for inclusion since they were more likely to have experienced several treatment lines and thus would be able to contribute to a discussion regarding a broader range of treatment modalities and effects. Furthermore, uncertainty among decision-makers (clinicians, patients, regulators, HTA/bodies, and reimbursement agencies) seems to be particularly present in the context of late-stage LC, due to the increasing amount of treatment options and treatment combinations for all stages of NSCLC, but especially for advanced stage LC (US Food and Drug Administration, 2020). Exclusion criteria were: 1) cognitive impairment or inadequate verbal skills that may render them incapable of agreeing to participate in an informed and voluntary fashion (as evaluated by the clinician); 2) inability to understand study materials (as evaluated by the clinician); 3) physical or psychological impairment that prohibits their participation in the focus group (as evaluated by the clinician). The clinical partners of both study centers made a primary selection of patients that could be contacted based on their health status and the inclusion and exclusion criteria. These patients were asked if they were interested in participating either during a visit to the hospital, by phone, or during hospitalization.

### Qualitative Approach and Data Collection

The qualitative study design involved four FGDs with between 5-7 LC patients per group (Durosini et al., 2021). FGDs were chosen as a method for data collection because they allow for interactivity between participants, active discussions guided by the researchers, and thereby may generate topics that researchers were previously unaware of (see Supplementary Appendix S1 for focus group guide and questions) (Durosini et al., 2021). FGDs were conducted by the authors of this paper (SP and SO in Italy and RJ, RA, and IH in Belgium), who have varying backgrounds (psychology, pharmacology, regulatory sciences, drug development) and experience with qualitative approaches and conducting FGDs. All discussions involved Dutch-speaking patients and Italian-speaking patients and were audio-recorded with participants’ consent.

Every FGD started with the same procedure; participants were given an information sheet, informed consent form, and a survey containing questions asking about their demographics. After each participant signed the informed consent, a FGD guide containing a series of open questions was followed (Supplementary Appendix S1) (Durosini et al., 2021). To increase procedural comparability among the discussions conducted in the two countries, both teams used the same discussion guide. When the moderator judged that the discussion on a specific topic had reached a point of saturation, a predefined list of potential treatment characteristics (Durosini et al., 2021) based on a literature review was read out loud as a way to spark further conversation. When no other new characteristics or insights emerged on a specific topic, the next question was asked. Participants’ health literacy was assessed using the Chew Brief Literacy Scale (Chew et al., 2004) and a short questionnaire was completed by the participants to gather information on socio-demographics.

### Data Processing and Analysis

The audio-recordings of all FGDs were transcribed ad verbatim in the original language and pseudonymized. Names were replaced by codes, and all other personal information not related to their treatment experience was removed. Transcripts were subsequently translated to English by a professional transcribing company (Lacey and Luff, 2007). Thematic analysis, as described in detail in Durosini et al. (2021), was used to assess the transcripts and notes from the FGDs. Data were analyzed using NVivo software (edition 12). The thematic analysis and focus group conduct used a mixed bottom-up and top-down approach. Specifically, the bottom-up (inductive) approach implied that patients were asked open questions about which aspects matter most to them. A bottom-up analysis was used to derive themes from their answers to these open questions. Conversely, a top-down (deductive) approach was done by asking about, and analyzing patients’ views on the side-effects and treatment outcomes associated with marketed and investigational drugs identified in the scoping literature review outlined in Durosini et al. (2021). The deductive analysis also considered the HRQL definition of the FDA to assess which aspects patients felt were important in determining their HRQL and how they could be categorized as physical, psychological, or social aspects. A combined bottom-up and top-down approach was taken in order to ensure the treatment aspects reflected in the themes are those that matter most to patients with respect to their HRQL, and inclusive of those side-effects and treatment outcomes of investigational and marketed drugs identified in the literature review that patients find important. Specifically, a bottom-up approach, i.e., deriving the themes directly via patients was taken to ensure that the themes were key in determining patients’ HRQL, plausible from the patients’ perspective, i.e., align with their experienced, lived disease and treatment experience. A bottom-up approach thereby helped avoid omitting potentially relevant treatment aspects included in the themes, and thereby avoid potential bias in the findings. In addition, a top down approach was taken as well, to ensure patients provide their views on potential “future” treatment outcomes and side-effects, even though they have not experienced them but could be definitive in determining their HRQL.

## Results

### Study Population

#### Belgium

In total, 12 stage III or IV NSCLC patients, contacted by the clinical partner of the university hospital in Leuven (Belgium), agreed to participate in the FGDs. The average response rate across the two FGDs was 56%, meaning that from all contacted patients 56% agreed to participate. Age characteristics were reasonably similar among the two FGDs with a mean age of 64.9 years [SD age: 6.82 years; age range: 52–78 years; 83% males; median age: 66.5 years; inter-quartile age (IQR): 6.8 years]. On average, there was a 2.8-years gap between diagnosis and participation in the FGD (mean age at diagnosis: 62.1 years; age range: 48–73 years). Using Chew’s three-item health literacy screening, all patients were coded as ‘moderate literacy’ (Chew et al., 2004). The majority of LC patients indicated to have as highest education: 1) a high school diploma (41,7%), 2) a bachelor diploma (25,0%), 3) no high school diploma (16,7%), or 4) a master diploma (16,7%). Nine participants (75,0%) reported that they were receiving LC treatment at the time of the FGD, with all patients not receiving treatment (*n* = 3) being concentrated in the first FGD. The most frequently taken treatments among Belgian participants were immunotherapy (42%), chemotherapy (17%), and a combination of chemotherapy and immunotherapy (8%).

#### Italy

A total of 12 NSCLC patients stage III or IV agreed to participate in the FGDs in Italy, and the response rate was 57%. Age characteristics were similar among the two FGDs with a mean age of 57.33 years (SD age: 8.56 years; age range: 42–72 years; 42% males; median age: 58.5 years; IQR: 13.5 years). On average, there was a 2.08-years gap between diagnosis and participation in the FGD (mean age at diagnosis: 55.25 years; age range: 41–68 years). Patients were coded as ‘moderate literacy’. The majority of LC patients indicated to have a high school diploma (41,6%), whereas 58% declared they did not finish high school. All 12 participants were receiving treatment at the time of the FGD. The most frequently taken treatments among Italian participants were immunotherapy (33%), chemotherapy, (17%), a combination of chemotherapy and immunotherapy (17%), and biological therapy (33%).

Across the four focus groups in Italy and Belgium, participants’ median age was 62 years (IQR: 9.3) and the average age was 61 years.

### Themes Capturing Key Determinants of Health-Related Quality of Life Among Lung Cancer Patients

Patients agreed on the importance of quality of life. Patients shared several personal definitions of quality of life and what it meant to them. Participants often reflected on quality of life in relation to a personal “endpoint”, where the benefits of the treatment would no longer outweigh the burden. For some participants, these endpoints were general: *“It has to be a life worth living.”* #FG2_A/Belgium, *“Try to live a life as normal as possible, as similar as possible to before.”* #FG1_C/Italy, and *“The expectation was to start getting my life back a bit. Professor XX also told me, “My task is to bring you back to life first.”* #FG1_D/Italy. Other participants had a clear description of which side-effects would bear such a decline in quality of life that they would stop undergoing treatment: *“I will undergo every treatment as long as my brain functions.”* #FG2_C/Belgium. Patients unmistakably expressed during the FGDs that they wanted to live longer but not at any cost, but how this “cost” was assessed and how big the range of “worth living” was, differed from patient to patient. It was clear though, that across both countries, LC patients’ quality of life was influenced by physical, psychological, and social aspects. Based on this, results of the FGDs are presented below according to the main themes identified through the thematic analysis of the focus group discussions: physical aspects, psychological aspects, and social aspects.

#### Physical Aspects Influencing Health-Related Quality of Life: Skin Conditions, Nausea, Fatigue, Risk of Infections, Sensory Abnormalities, Pain, and Changes in Physical Appearance

##### Skin Conditions

Several patients reported an undeniable itching feeling, especially as a side effect of immunotherapy, on diverse parts of their body resulting in the urge to scratch. This itching feeling was persistent, causing patients to continue to scratch their skin until it bled: *“That’s a side-effect of the immunotherapy, you start to itch, then I begin to scratch it, but you keep scratching till you get through it.—Till you get through everything, till you bleed.”* #FG1_C/Belgium + #FG1_G/Belgium, “*(My) nails were breaking, I was getting cuts. I said to my son* *“But how can I go on like this?”” #*FG1_E/Italy, and “*The other one (pill) really killed me. In the first few months I lost all of the skin from my hands, face, spots.”* #FG2_B/Italy. This itching feeling was debilitating for them since this did not allow them to focus on other activities: *“It’d drive you mad, really.”* #FG1_G/Belgium, and *“Essentially, the fact that I was losing my skin, my hands were bleeding… how can I live like that? Better to die…”* #FG2_B/Italy. Other patients also had some skin conditions like a rash or burns from chemotherapy or radiation therapy, which together with the bleeding/peeling skin negatively affected patients body image (as further described in “Changes in Physical Appearance”).

##### Nausea

The theme of nausea emerged in both countries and was experienced by the majority of participants. Nausea and also vomiting in some cases were highly related to chemotherapy: *“what you experience with chemo, the nausea.”* #FG2_C/Belgium, *“a sense of nausea… iron, it was like I had iron inside me, rust.”* #FG2_B/Italy, and *“I couldn’t even manage to take (name of the drug), the side effect was fainting, or almost. It was continuing and continuing vomiting and nothing ever came up because I wasn’t able to hydrate myself.”* #FG1_B/Italy. Nausea had a significant effect on the participants’ possibility to live a qualitative life because, firstly, they could sometimes no longer engage in some activities because of the nauseous feelings: *“I had nausea and you’re a different person.”* #FG2_A/Belgium and the nausea could not be helped by any medication: *“I was in bed and I remained weak. Then at a certain point they took me to the emergency room, I spent a night there, because I could not manage to take things (medication) to normalize the situation.”* #FG1_B/Italy, and *“Chemo is devastating… it kills you… you are dead, for 4 months it is like being dead.”* #FG2B/Italy. Secondly, the feeling was a constant reminder of their cancer. *“Yes, you have a lot of side effects, such as nausea, that remind you of it.”* #FG1_A/Belgium. In addition, nausea impedes patients to have a normal family life: *“When I need to prepare food I feel sick. When I need to start preparing a meal for my husband and my daughter, not only am I not hungry but it actually disgusts me.”* #FG1_E/Italy. Another patient even needed to quit his therapy because of nausea: *“They brought me to the emergency room because I could not eat any more, I vomited twenty times, after two times (receiving chemotherapy), I had to stop it because it was highly toxic…”* #FG1_B/Italy. One patient explained that he had experienced chemotherapy more than 30 years ago and voiced that he felt that the management of nausea related to chemotherapy had already improved significantly over the years: “*Yes, the medication has improved, yes. There’s no doubt about it. Compared with before, I got chemo then for an entire year. There wasn’t much to smile about. (…) at the moment itself you had to throw up, there wasn’t enough medicine at the time to prevent it.”* #FG2_B/Belgium.

##### Fatigue

Immunotherapy and chemotherapy were two treatment modalities that were reported to cause fatigue, so intense and exhausting that it hampered patients in performing any physical and psychological activities: *“Yesterday I was watching Grenslanders with my children, I saw the first 10 minutes and then I was out.”* #FG2_A/Belgium, and *“With chemo I was so tired, (…), I have always walked, I have climbed so many stairs and steps in my life and, conversely, since I have had chemo, I have found it difficult to walk, to cook, I was making junk food, my poor husband who was used to eating well… I wasn’t able to make more than that (…) I would say to these people who make these drugs to try to cut that out because otherwise your quality of life is impacted.”* #FG1_F/Italy. When the subject of fatigue came up, it was noticeable that everyone had a story to tell. The fact that everyone could recognize this particular side effect and that they were not the only one going through this made them feel better: *“I’m soooo tired. I could sleep all day long, and I’m happy because she’s got that too.”* #FG1_G/Belgium. The expectations of patients concerning their physical activity varied, ranging from *“I might just lie down for half an hour and I’ll be right as rain. But after two or 3 hours I’m shattered again.”* #FG1_G/Belgium to *“I get tired earlier, but I can easily go walking for half a day, Nordic walking but I’m really tired afterwards.”* #FG1_D/Belgium.

##### Risk of Infections

Lower immunity was mostly discussed in terms of the risk of getting an infection. Lower immunity was seen as being detrimental in two ways. First, it increased patients’ likelihood of getting infections: *“I also got two infections in my big toe, for which the doctor had actually sent me to the cosmetologist, they had to suspend use of the drug for 2 weeks because there was this infection in this big toe and then it got better and there was nothing more.”* #FG1_E/Italy. Second, it prevented patients from participating in some activities, thus limiting their freedom: *“I can still remember when my grandson had just learned to walk, he came over to me and they all said ‘no don’t, don’t, you have a cold, you have to stay away'.”* #FG2_D/Belgium, and *“Your immune system is at its lowest now. Don’t stand near sick people in the shop, stay away from the bakery, because if there’s someone there who has something, you’ll get it too.”* #FG1_B/Belgium.

##### Sensory Abnormalities

The theme of sensory abnormalities relates to a multitude of side effects including: taste differences, tingling, hearing impairment, and different perception of temperature. Firstly, taste differences were observed both in food as in drinks and were associated with chemotherapy*: “I enjoy drinking coke, and I couldn’t drink coke anymore because the only thing I could still taste in the cola was the citric acid.”* #FG1_C/Belgium, *“The flavour of food too.”* #FG1_A/Belgium, *“I don’t have the pleasure of food.”* FG2_C/Italy. Secondly, tingling was another side effect, which was the result of nerve damage caused by radiation or chemotherapy. The participants that experienced this were all annoyed by it but were able to put it aside for the greater goal: *“My feet tingle because the nerves are dying because of the chemo. It’s really annoying, but you learn to live with it don’t you.”* #FG1_B/Belgium. Thirdly, one patient was faced with hearing loss as a result of his treatment. The possibility of no longer being able to hear was too much for him. Other participants were also convinced that they would stop the treatment if it would make them lose their hearing: *“But that was the choice, did I want to go deaf or did I want—well yes, that wasn’t an option for me.”* #FG1_F/Belgium.

##### Pain

Pain, both due to the cancer and the therapy, was another category of issues that was found to negatively influence quality of life. Participants extensively discussed pain caused by the therapy: *“No, no, I was crying every night but not because of the cancer, because of the pain.”* #FG1_D/Italy. Other patients described pain as due to the metastasis of the cancer. One participant for example described severe headaches due to the brain metastases: *“I get headaches and I can’t take anything for it. (…) I can eat them like candy.”* #FG1_C/Belgium. One participant with several metastases with one being bone metastases went through a lot of pain: *“Bone pain is something else, that’s very intense pain.”* #FG2_C/Belgium. This patient stressed the fact that some cancers cause pain that even the most potent painkillers cannot alleviate. This pain was so intense that one could become dependent on pain medication: *“Pain medication (…) in the room um, X percent of the patients couldn’t wait their turn anymore and were really almost aggressive, hysterical, because of the pain.”* #FG2_C/Belgium. The majority of the participants noted, however, that they had not experienced such severe pain. Nonetheless, they were all convinced that they were a select group since they had met several patients over the years who had encountered excruciating pain. Notwithstanding, some patients suggested that they would bear pain in exchange for seeing the cancer stop growing: *“I would accept being bed-bound, having nausea, pain in my legs, and maybe I would also accept that I may not completely recover, if I were sure that it (the cancer) would stop growing.”* #FG1_C/Italy, and *“In any case the moral of the story is that today I would do everything that I have done again, despite all the pain.”* #FG2_B/Italy.

##### Changes in Physical Appearance

Patients highlighted that bodily changes, caused both by the cancer and LC therapies, made them insecure about their body image, and also negatively impacted how they interacted with others (as further described in “Social Interaction”). Specifically, patients stated that excessive changes to their body weight would reinforce and publicly make the idea of being an ill person: *“Yes, excessive changes to the body are always linked to the issue of quality of life. The fact that… it is as if I don’t want others to see that I am unwell because it is also a way for me to be stronger. If other people treat me like a normal person, pass time with me, I feel stronger.”* #FG1_A/Italy, and *“I am not eating much and I get angry because in other people’s eyes it looks like I am eating whole roast chickens myself, and yet that’s how it is… I put on all these kilograms and I do not know why.”* FG1_E/Italy. Weight gain is also an issue because sometimes people around patients seem to blame him/her: *“My sister tells me ‘But look how swollen you are.' But what can I say? She thinks I eat a lot, but I don’t.”* #FG_B/Italy. Hair loss, particularly associated with chemotherapy, seemed to be a side effect that did not bother all patients in the same way. Whether or not hair loss was acceptable seemed to depend on the severity of their hair loss. Whereas for some the experienced hair loss was acceptable: *“The hair (loss) is alright, it’s nothing.”* #FG1_C/Italy, the idea of losing all hair, would be: *“Devastating on the personal level.”* #FG1_B/Italy for other patients. Other participants did not experience hair loss as a side-effect of their NSCLC chemotherapy.

#### Psychological Aspects Influencing Health-Related Quality of Life: Autonomy, Freedom and Independence, and Uncertainties Regarding Patients’ Future Health State

Patients were very vocal on the psychological effects of cancer. Most of the patients actively sought for the positive aspects and tried to remain hopeful for the future, portraying a “fighter’s mentality”: *“You have to go, keep working, you have to keep morale up.”* #FG1_C/Belgium, *“I’m only going to go crawl into a corner, sit and cry, when the professor comes and says: we don’t have anything else for you anymore. Then you still have time to mourn.”* #FG2_A/Belgium, and *“I react and make efforts (…) in any case your life changes in a moment. (…) you need to change your lifestyle, you need to change and reset everything a bit, and gradually you get used to it… I am doing quite well now.”* FG2_A/Italy. In general, participants receiving immunotherapy at the time of the FGDs seemed quite happy with their health status. One of the things that was not always put into words but became visible, was the pleasure they felt when others related to their story. Knowing others were going through the same process and face the same negative aspects created a connection between patients: *“You get to know the other people, you get more sociable, you share your problems with one another…”* #FG1_G/Belgium. Several patients showed interest in connecting through patient organizations with people who had had similar experiences.

##### Autonomy, Freedom, and Independence

While someone found it important to *“still go on holiday”* #FG1_F/Belgium, others found meaning in *“going back to work”* #FG1_G/Belgium or *“riding my bike”* and “*going to the vegetable garden”* #FG2_B/Italy. Someone else emphasized the need to go back to a normal life like before having cancer and receiving therapy at the hospital: *“I had radiotherapy, five applications and then finally I said I’m going on holiday for a while.”* #FG1_F/Italy. Sometimes patients chafed at the realization that they were less independent and free as before: *“I find it really difficult to get out of it. My sister comes, she takes me out, but I don’t want to go out, I don’t want to see people and this is a terrible lifestyle for someone who has this type of disease. My daughter’s father in law calls me and tells me ‘Come here so we can chat’ and I don’t want to chat, ‘come here so we can go out, let’s go out for a walk’ and I don’t want to walk.”* #FG1_E/Italy because the treatment regimen deprives them of their independence: *“If I need to take a drug every 15 minutes, how can I manage my daily life?”* #FG1_E/Italy.

Several patients stressed the importance of being able to be professionally active. One patient found great joy but also meaning in her work, and by losing her work, she found that she had lost a big part of her life: *“Sitting about at home isn’t easy if you’ve always worked. I mean it’s really driving me mad.”* #FG1_G/Belgium. On the other hand, someone else complained about the duality between still having all his duties but being deprived of the things he enjoyed: *“I’m still well enough to go working and to do whatever else, but there are two things that I can’t do. One is that I can’t drive my own car, and the other is that I can’t fly. So, I can’t go on holiday.”* #FG1_C/Belgium. This restriction on personal freedom, including not being able to drive a car, emerged as an important factor, one mentioned by every participant: *“You’re not allowed to drive anymore? I’d hate that.—Yes, it’s a restriction, not a bodily restriction but it’s…"* #FG1_F/Belgium + #FG1_B/Belgium. This was mentioned to be linked to staying socially active; being homebound limited their ability to connect with others and to maintain social interactions. Patients further stated that being confined to bed, as a result of a therapy, with a consequent loss of independence and autonomy could be one of the most problematic side effects of a therapy. Further, while they considered it acceptable to be in need of *“perhaps a little help”* and, for a limited time, they clearly *“would prefer to be independent”* #FG1_B/Italy and feared being bed-bound for an indefinite period of time.

##### Uncertainty Regarding Their Future Health State

As participants were all in an advanced stage of LC, they were aware of the fact that LC may very likely be deadly for them, and this faced them with the constant fear of how much longer they had to live. On several occasions, they reflected on the fact that this created a situation with a lot of uncertainty for patients and their relatives. They recognized that the path might be long: *“you don’t recover straight away… it takes a bit of time, it takes some years, at least that is the case for the experiences that I have encountered up until now.”* FG2_A/Italy. They often feel uncertainty related to the time they had to live: *“I think that what I wanted to know was to try to understand how long I had left to live, nobody told me that… alright, there must be rules, you can go on for between one and five (years), or you will die a natural death.”* #FG2_B/Italy. In addition, the uncertainty whether their health status will stay the same for a more extended period of time made it hard for patients to engage in long term projects: *“Long term there’s not much anymore.”* #FG1_B/Belgium, *“I live day by day. Every day is gold for me.”* FG1_C/Italy. Many of the participants believed that the moment of diagnosis, one’s long-term plans drifted away and were replaced by thoughts of one’s funeral and estate planning. This causes a difficult situation for both the patient and his/her partner since they can no longer plan ahead anymore. Also, the question of whether the medication will continue to work, whether they will still be able to get the medicine after their study ended, raised concerns among participants: *“It’s so new and long term, will it keep working, will it stop?”* #FG1_F/Belgium and *“What do I expect if this does not work? Well… I am becoming a grandmother and I need to know if I will see my grandchild next year!”* #FG1_B/Italy. These questions created uncertainty resulting in patients suffering and worrying: *“Everything’s always maybe, maybe, maybe.”* #FG1_E/Belgium. At a beginning of a care path, some patients express their feelings of being invincible: *“This genetic mutation, here at the hospital they immediately gave me this drug and, at the start, I felt strong with this drug, for me it was also a positive way to react, I immediately had the impression that this drug was working very well.”* #FG1_C/Italy, whereas these feelings decline when the therapy does not work anymore or in case of relapse: *“Yes, that’s right, day by day it is OK, but I am a little more negative than before because before I had a bit more hope and, seeing that it is not going well one moment I am quite… I had surgery 2 years ago, not even one and a half years, it relapsed.”* #FG1_B/Italy. One of the things patients perceived as a kind of certainty and peace of mind was the knowledge that if the current treatment option would fail, other treatment options were still available: *“If there’s a setback, we still have !ve or six other options.”* #FG1_G/Belgium. Others who did not know whether other medication would become available faced much more uncertainty: *“You’re sitting on the joyride that is science, and you just have to hope it’ll keep moving.”* #FG2_A/Belgium.

Those patients who were satisfied with his or her health status at the time of the FGD did not want to receive negative news that might disturb their delicate psychological equilibrium. Every patient had to go to the scanner every once in a while. This moment was generally marked as a moment of considerable uncertainty. The scanner gave them an update on how they were doing and could possibly drastically affect their lives: *“I had to wait 5 months for the results of a scan. That’s too long for me. (…) there’s too much uncertainty.”* #FG1_G/Belgium, *“I have a CT scan in 1 week. For me, that is the most tragic moment because I live with incredible anxiety.”* #FG1_C/Italy, *“At the start you needed to have a scan every 2 months. Now it’s every 3 months, but you still feel tense, you know.”* #FG2_B/Belgium. Multiple patients noted that besides for them, this was also a heavy psychological burden on their partner and other relatives: *“Then my wife will say—well, she’ll start worrying. Especially because my scan date is getting closer.”* #FG2_D/Belgium.

#### Social Aspects Influencing Health-Related Quality of Life: Social Interaction and Communication With Healthcare Providers

##### Social Interaction

Patients identified their personal situation as an important factor influencing their attitude, behavior, and assessments towards treatment options and compliance. In particular, family composition affected nearly all participants; the presence of a partner, children, grandchildren, gave participants a reason to live and fight: *“I thought, I’ll never see the little man grow up. He’ll be six in December. That’s a gift that I’ve been given and that I’m very grateful for.”* #FG2_D/Belgium, *“I am becoming a grandmother and I need to know if I will see my grandchild next year.”* #FG1_B/Italy, and *“I have a daughter now, who still lives with us, the other daughters are married, grandchildren, I am starting to collect them from school again.”* #FG2_D/Italy. On the other hand, some patients considered family to be a reason to stop treatment. Two participants said they would rather stop treatment and terminate their life than to put their family through a lot of suffering. The first participant watched her mom deteriorate and did not want her children to remember her that way: *“I watched her waste away and I said: ‘No, I don’t want to put my children through that.”* #FG1_G/Belgium. The second participant applied a more general principle; he did not want his relatives to perceive him as a burden: *“As long as I’m not a burden to someone else then yes, I’ll go through with it.”* #FG2_E/Belgium.

Although patients described interacting in social activities as one of the favorable aspects, the effects of cancer and treatment can hinder their ability to participate. Several patients reported that they did not feel comfortable going outside and meeting people because of their altered looks. Hair loss and weight fluctuations (see also ‘Changes in Physical Appearance’) were the two changes participants named as having the most significant effect on how they felt about themselves and how people perceived them: “*I lost my hair, I lost 12 kilos, I had a huge moustache, that’s gone (…) you know what it's like, they say ‘hey hello’ and then ‘oh did you see him? He really didn’t look well did he?”* #FG1_A/Belgium and *“Perhaps I should not go out… when I go out, I do not go out to gain people’s compassion, because I hate the “Poor thing…“, I need to go out with a smile because if not they will say “Look how pale she is”, look how she is, look…”* #FG1_B/Italy. The idea they would have to face the gossip and the confrontation with others scared them. On the other hand, participants who did not have any visual side effects criticized that they weren’t seen as a cancer patient and were subsequently not recognized as “having a hard time’”. The fact that people did not see it made them feel their illness was not as valid as someone with visible symptoms or side effects. Another participant felt he should keep his cancer a secret to people around them, since telling them would be detrimental for their social interactions. When people know you have cancer, they become scared to visit you or to say something wrong: *“I hide it, the fact I have cancer. (…) Because if you tell them, people don’t know what to say to you.”* #FG1_B/Belgium. In conclusion, all participants agreed they wanted their social interactions to remain as much as possible unchanged despite their cancer.

##### Communication With Healthcare Providers

Patients spoke about the positive and negative moments of communication with healthcare workers. The most prominent negative feedback patients gave was that they felt the hospital was too big, which made it difficult to have a good flow of information.—*“The hospital is far too big. That there isn’t enough interaction.”* #FG2_A/Belgium, and *“The hospital really is like a factory.”* #FG2_C/Belgium. Because the hospital is too big, patients had to repeat their concerns and problems several times before action was taken. Participants attributed this lack of responsiveness to the fact that the high number of different staff (both referring to different types of healthcare professionals and a high turnover in the hospital staff) hampered efficient communication between healthcare workers and patients: *“I showed my edema to one of the people running the study here and yes she said: ‘that could be one of the consequences and that can be burdensome’. But there was no reaction to try and work on it anymore until I saw XXX again, “Yes” she said, ‘Why didn’t you come down here to the edema department?”* #FG2_A/Belgium. The most vocal participants noted that the way you got treated depended highly on the assertiveness of the patient. Surprisingly, patients involved in a clinical trial reported experiencing better communication, although these patients also went to the same hospital. However, when the study ended, they encountered the same experiences as the patients not involved in a clinical trial: *“The only thing I think is a pity about it (the clinical trial) is (…) after the study was finished. Then you are kind of abandoned, left to your fate.”* #FG1_G/Belgium. Despite the limited flow of information, patients stressed the fact that healthcare workers were always friendly and that their motivation and how they interacted with patients sparked joy in difficult times.

The opinions on the exchange and availability of information were highly divided. One patient felt that *“physicians don’t like giving bad news”* #FG1_B/Belgium although he would rather get all possible information to prepare himself. Another patient, however, did not share the same experience: *“They explained properly to me what the effects would be and how long it would last.”* #FG1_E/Belgium. One patient, however, noted that he believed that receiving too much information was burdensome, and he would rather not know. The information was often too technical and not interpretable by a layman: *“You don’t understand what you are looking at if you saw your blood results. Just lots of figures.”* #FG1_F/Belgium. About half of the participants were eager to receive additional information on how to live with LC and LC itself. They requested they would be updated on new clinical possibilities and potential future treatments: *“I’m curious about the new medication you know. But yes, we’re going to have to wait.”* #FG1_A/Belgium. They felt that the available information was too limited, and their attempts to gain knowledge were discouraged by doctors: *“As soon as you start talking about for example cannabis oil here in the University Hospital, they say sorry, that’s not what we do.”* #FG2_D/Belgium.

Participants spoke about delivering terrible news to patients in a manner considered inappropriate by themselves: *“How long will I need to take this pill?”, and he (the doctor) told me “Ah, madam, for your entire life!”* #FG1_E/Italy and *“in my ignorance I said: 'But can’t I continue the immunotherapy?' And he (the doctor) looked at me and said ‘Madam, do you want to die?' But he said it in a provocative manner and I remained quiet”* #FG1_F/Italy. A participant received bad news on the phone: *“They called me to tell me on the phone that they’d operate on my head. Like that, on the phone.”* #FG1_C/Belgium. He regretted this since after this call he had had many questions and couldn’t sleep because of the stress it caused him. Another participant did not receive bad news himself but remarked based on his experience observing other people receiving bad news: *“As an outpatient, you’re all in that circle, in the circles. Then they close the curtain and the professor comes and tells you some bad news!”* #FG2_A/Belgium. Not only would he not appreciate receiving bad news this way himself. Seeing and hearing others who have the same cancer receive bad news causes emotional distress to the surrounding patients as well.

## Discussion

This study reveals in-depth insights into how LC patients perceive their HRQL and the factors that are most impactful in determining their HRQL. In particular, LC patients prioritized aspects related to physical, psychological, and social aspects influencing HRQL. Within the category of physical aspects, patients highlighted the following symptoms and side-effects: skin conditions, nausea, fatigue, risk of infection, sensory abnormalities, pain, and changes in physical appearance. Among the psychological aspects, patients discussed autonomy, freedom and independence, and uncertainties regarding their future health state. Finally, patients highlighted the importance of social interaction and communication with healthcare providers related to the theme of social aspects.

Gaining a better understanding of how LC patients perceive the ways their HRQL is affected by their illness and therapy may aid patient-centric decision-making across the drug life cycle, by providing stakeholders (drug developers, regulators, reimbursement bodies, and clinicians) insights about the treatment aspects of importance to LC patients as well as the unmet needs LC patients may have regarding available treatments. In particular, aspects of importance to patients identified in this study may inform drug developers about unmet treatment needs not resolved by all available therapies; findings from this study may trigger pharmaceutical companies to develop treatments aiming to avoid or resolve skin conditions, nausea, fatigue, risk of infections, sensory abnormalities, and pain. Of note, treatments for some of these side-effects already exist. For example, while patients considered nausea a disabling side-effect, medications for nausea already exist and are being prescribed, such as Netupitant Palonosetron for the cisplatin/carboplatin schemes on the day of treatment and Alizapride Hydrochloride to be taken at home. Efforts towards a better management of reported problems are also increasing. In the individual treatment context, patients may be encouraged to communicate about their problems and ask healthcare providers (such as their treating oncologist, nurses, oncocoaches) for personal advice for the management of these problems.

However, while acknowledging the existence of treatments for some of the reported issues, for other issues reported by patients, such as fatigue and excruciating pain, our results indicate that presently no treatments are available. Likewise, while recognizing increasing efforts towards improved management and communication of patients’ experienced physical and psychosocial side-effects and symptoms, our results highlight that patients in clinical practice are still confronted with both physical and psychosocial issues that require further support. Hence, results from this study argue in favor of a continued and expanded consideration of patients’ reported side-effects and symptoms in order to improve LC patients’ quality of life. If patients are given the opportunity to ask for help and advice, symptoms and side-effects are likely better managed. Examples of efforts that should be continued and expanded, based on patients’ individual needs, include the systematic incorporation of staff trained to support patients from a psychosocial viewpoint (such as psychologists, oncocoaches, social workers), and the expanded use of tools (such as a symptom diary) that assesses patients’ physical and psychological burden related to LC and gives healthcare professionals the opportunity to manage the patients’ reported problems.

This study also underscores the importance of increasing communication, awareness, and consideration related to the psychological problems of LC patients. Specifically, several patients highlighted during the discussions uncertainties about their future health state. This is likely due to the difficulty healthcare professionals encounter in giving a correct individual prognosis, as it depends on several patient characteristics such as symptom burden and treatment compliance. Patients experiencing uncertainties will likely benefit from the development of solutions to help relieve their uncertainties, such as ways to improve communication towards these patients about the long-term expectations regarding treatment outcomes. For example, based on patients’ individual information needs, ways to improve communication with such patients about their long-term treatment results and side-effects, and methodologies supporting clinicians and patients, such as shared decision-making tools, could help address these patients’ uncertainties in the individual treatment decision-making context. Patients who are informed about the side effects they may experience in the future will likely be more therapy compliant and this may in turn positively impact their life expectancy. Another solution to support LC patients from a psychosocial viewpoint, that does not require additional time from healthcare providers, may be (online) LC support communities; based on qualitative interviews with advanced LC patients, Walsh and Al Achkar (2021) explore the value of online LC support communities to provide support, camaraderie, and specialized health information.

The importance of capturing and including HRQL data in LC drug development and treatment decision-making has been highlighted by several previous empirical and literature studies. Particularly, the categories identified in the present study related to physical, emotional, and social impact on HRQL were also revealed in a qualitative interview study by Rowland et al. (2016). Regarding psychological aspects, He et al. (2019) examined the role of LC patients’ mood in influencing their HRQL and concluded that interventions that facilitate adaptive coping, reduce negative mood, and enhance positive mood could help to improve or maintain HRQL in patients with advanced LC. Further, Franceschini et al. (2020) retrospective observational cohort study among stage IV LC patients highlight the negative impact of weight changes. Schmidt et al. (2016) conducted a systematic review on LC preference studies and revealed the negative impact of nausea and vomiting on patients HRQL. Likewise, the importance of psychological support and management of uncertainties among LC patients is highlighted by Kurita et al. (2013); suggesting that both during and after treatment, individuals with LC may experience a difficult disease course with higher levels of distress related to physical symptoms, greater challenges in psychological health and daily living, and higher levels of burden from their symptoms than those with other types of cancer.

The physical and psychological aspects influencing HRQL identified in this study may also inform future clinical trial design in LC. Specifically, the identification of clinical trial endpoints as well as the further development of existing patient-reported outcome measures could be broadened to include the physical and psychological side-effects and symptoms of importance to patients highlighted by LC patients in the present study. Several studies have investigated the impact of (novel) NSCLC treatments on HRQL. HallSinghal et al. (2019) performed a systematic review to examine *Patient reported outcomes* (PROs) and HRQL among cancer patients receiving immunotherapies and revealed that few randomized studies reported PROs and patient HRQL data. They also conclude that currently used instruments likely fail to capture important aspects unique to such novel therapies (such as psychosocial aspects related to the disease and treatment) and underscore a need for PROs that are inclusive of these aspects. Likewise, King-Kallimanis et al. 2019 investigated PROs in clinical trials of anti-PD-1/PD-L1 inhibitor immunotherapy, which was a trial submitted to FDA, and conclude that the PRO data collected did not consistently assess important symptomatic events. Bennett et al. (2017) performed systematic review to explore the impact of SCLC on HRQL and the PROs used to capture this impact and conclude that paucity exists regarding the reporting on NSCLC HRQL outcomes. Likewise, Van Der Weijst et al. (2019) performed a systematic literature review of clinical trials in NSCLC and conclude that while reporting HRQL data is important to support clinical decision-making as well as marketing authorisation and reimbursement decisions, the methodology of reporting HRQL remains poor. Among the instruments currently available to measure quality of life, primarily the EORTC QLQ-C30-LC13 (EORTC, 2018), but also the Functional Assessment of Cancer Therapy-Lung (FACT-L) Scale (FACIT, 1995) and its LC specific subscale FACT-LCS (FACT-LCS, 1995) are the most commonly used instruments in LC clinical trials (HallSinghal et al., 2019; King-Kallimanis et al., 2019; Van Der Weijst et al., 2019). Comparing our results to current LC specific scales (EORTC QLQ-C30-LC13 and FACT) reveals that the following side-effects and symptoms, observed in our study, are not included in all presently used HRQL specific LC scales: 1) skin conditions, 2) risk of infections, 3) sensory abnormalities, 4) increased weight, 5) autonomy/independence, 6) uncertainties regarding side-effects and (duration of) positive treatment outcomes, and 7) communication with healthcare providers. The identification of these aspects, such as psychosocial impact of the latest cancer therapies, skin conditions, increased risk of infections, and sensory abnormalities is likely due to the fact that patients participating in the discussions were taking novel treatments (immunotherapies) with novel associated side-effects and uncertainties.

Aligning with conclusions yielded by previous researchers (Bennett et al., 2017; Bouazza et al., 2017; HallSinghal et al., 2019; King-Kallimanis et al., 2019; Van Der Weijst et al., 2019), our findings also argue in favor of systematically including and reporting HRQL instruments and outcomes in LC drug development, regulatory decisions, and clinical shared decision-making to assess and understand the experience of LC patients. Our findings also argue in favor of broadening and updating current HRQL instruments to be inclusive of NSCLC symptomatology and side-effects related to novel (immune) therapies. Further, whereas HRQL scales investigate patients’ *quantified* experience with these HRQL aspects, the present study also reveals the added value of incorporating *qualitative* research with patients to understand why these HRQL aspects are important in influencing patients’ HRQL and how they specifically impact patients’ lives.

This study demonstrates the value of qualitative research with patients as treatment end-users to understand their experience with their illness and treatments. The use of FGDs with open questions enabled to be as inclusive as possible by obtaining both broad and in-depth information on LC patient preferences. Moreover, it allowed seeking answers and clarification to sensitive questions without overburdening patients. Further, interaction between participants with varying treatment and disease exposure ensured a range of symptoms and side-effects across different therapies were revealed, thereby identifying HRQL factors important for patients along the LC spectrum, inclusive of aspects related to varying treatment and disease experiences. Finally, several researchers with varying backgrounds (psychology, drug development, clinical background) were involved in the conduct, analysis, and interpretation of the discussions, ensuring a relevant and correct interpretation of the data. This further allowed us to interpret the data accurately and avoid personal bias.

As for the limitations, it is important to note that the results of this (and other qualitative research) are dependent on the specific time, (individual) drug therapy context, as well as the type of participants included. Therefore, the results need to be interpreted considering the specific time period and context the study took place as well as in view of the type of participants that took part. Patients had to be physically and mentally able to participate in the discussion, and hence, it remains unknown whether patients that were not physically or mentally able to participate would have raised additional aspects in relation to their HRQL. However, even though we did not include patients physically unable to participate, the study results reveal the detrimental impact of being homebound, and limitations in LC patients’ autonomy and independence. Likewise, the importance of psychological aspects influencing HRQL was captured, even though participants who were mentally unable to participate were not included in the discussions. It is also important to note the influence of the individual drug therapy experience of participants on the identified symptoms and side-effects. In the results, we did not differentiate the results according to drug therapy as the goal was to provide an overarching view of important themes to patients across therapies and how they relate to their HRQL. However, it is worth noting that the identified side-effects are often specifically linked to certain therapies. Hence, different treatments (e.g., immuno-vs chemotherapy) will likely differently impact patients’ HRQL. For example, while novel immunotherapies are likely linked to fatigue, skin conditions, sensory abnormalities, and psychological uncertainties among patients, chemotherapy is likely linked to nausea, and changes in physical appearance. Another limitation relates to the presence of more dominant speakers in the discussions who went off-topic by telling detailed personal stories and thereby reduced the opportunity for other participants to contribute to the discussion. This was however minimized by the intervention of the discussion moderator experienced in the conduct of FGDs, who was able to involve also more shy participants and engage them in the discussion. Finally, qualitative research with social interactions is researcher-dependent and this likely influenced the narrative of the discussion. This was however counteracted by the involvement of multiple researchers in the conduct and analysis of the discussions. Furthermore, researchers across Italy and Belgium used the same focus group guideline to structure the discussion and ensure the same questions were addressed in each discussion.

## Conclusion

This study demonstrates that all aspects of HRQL are salient concerns for LC patients in stage III and IV, including physical, psychological, and social aspects. A better understanding of how LC patients perceive their HRQL and how it might be affected by different LC therapies should inform drug developers, regulators, reimbursement bodies, and clinicians about the treatment and disease aspects of importance to LC patients as well as the unmet needs LC patients may have regarding available treatments. Future efforts across stakeholders are needed to translate and incorporate these findings into the development, approval, and reimbursement of drugs that are successful in addressing the symptoms and side-effects that are detrimental to patients’ quality of life. Finally, this study underscores a need for individual treatment decision-making that is considerate of uncertainties among LC patients about their future health state, and ways for improving communication between healthcare providers and patients to do so.

## Data Availability

The datasets presented in this article are not readily available because they contain information that could compromise interviewees’ privacy and consent. Further inquiries should be directed to rosanne.janssens@kuleuven.be.
